# Demographic and behavioural correlates of energy drink consumption

**DOI:** 10.1017/S1368980022001902

**Published:** 2023-07

**Authors:** André O Markon, Ming Ding, Jorge E Chavarro, Beverly J Wolpert

**Affiliations:** 1 Center for Food Safety and Applied Nutrition, US Food and Drug Administration, Office of Analytics and Outreach, Division of Public Health Informatics and Analytics, Harvey W. Wiley Building (CPK 1), 5001 Campus Drive, 2C-103, College Park, MD 20740, USA; 2 Department of Nutrition, Harvard T.H. Chan School of Public Health, Boston, MA, USA; 3 Channing Division of Network Medicine, Department of Medicine, Brigham and Women’s Hospital and Harvard Medical School, Boston, MA, USA; 4 Department of Epidemiology, Harvard T.H. Chan School of Public Health, Boston, MA, USA

**Keywords:** Energy drinks, Risky behaviour, Epidemiology

## Abstract

**Objective:**

Energy drinks are consumed for a variety of reasons, including to boost mental alertness and energy. We assessed associations between demographic factors and various high-risky behaviours with energy drink consumption as they may be linked to adverse health events.

**Design:**

We conducted cross-sectional analysis including basic descriptive and multivariable-adjusted logistic regression analyses to characterise demographic and behavioural factors (including diet quality, binge drinking and illicit drug use, among others obtained via questionnaires) in relation to energy drink consumption.

**Setting:**

We used data from two large US-based cohorts.

**Participants:**

46 390 participants from Nurses’ Health Study 3 (NHS3, *n* 37 302; ages 16–31) and Growing Up Today Study (GUTS, *n* 9088, ages 20–55).

**Results:**

Of the 46 390 participants, 13·2 % reported consuming ≥ 1 energy drink every month. Several risky behaviours were associated with energy drink use, including illegal drug use (pooled OR, pOR: 1·45, 95 % CI: 1·16, 1·81), marijuana use (pOR: 1·49, 95 % CI: 1·28, 1·73), smoking (pOR: 1·88. 95 % CI: 1·55, 2·29), tanning bed use (pOR: 2·31, 95 % CI: 1·96, 2·72) and binge drinking (pOR: 2·53, 95 % CI: 2·09, 3·07). Other factors, such as high BMI, e-cigarette use and poor diet quality were found to be significantly associated with higher energy drink consumption (*P* values < 0·001).

**Conclusions:**

Our findings show that energy drink consumption and high-risk behaviours may be related, which could potentially serve as not only as a talking point for providers to address in outreach and communications with patients, but also a warning sign for medical and other health practitioners.

Energy drinks are consumed for many purposes, including boosting energy, improving mental alertness, enhancing athletic performance, satiating thirst and satisfying taste. Caffeine is especially common in these beverages, which may also contain various other ingredients, such as taurine, ginseng, herbs and amino acids^(1)^. Energy drinks are often sold in ready-to-drink formats (cans and shots) and classified as conventional foods/beverages or dietary supplements. Analysis of data collected in the 2003–2016 cycles of the National Health and Nutrition Examination Survey (NHANES), which is designed to be representative of the non-incarcerated population in the USA, found that 1·4 % of teenagers (ages 12–19 years), 5·5 % of young adults (ages 20–39 years) and 1·2 % of middle-aged adults (ages 40–59 years) consume energy drinks^(1)^. Sales of these drinks, including shots, in the USA are estimated to exceed $12 billion per year^(2)^.

Despite intake across age groups and anticipated sales growth in the USA, studies have shown that energy drink consumption may be of public health concern, particularly among vulnerable groups such as children and young adults^(3,4)^. Previous analysis of adverse events reported to the US Food and Drug Administration (FDA) Center for Food Safety and Applied Nutrition’s Adverse Event Reporting System (CAERS) and exposure calls to the American Association of Poison Control Centers (AAPCC) National Poison Data System (NPDS) found consumption of energy drink products were correlated to adverse events including tachycardia, nausea, vomiting, as well as nervous system and gastrointestinal system alterations, among other negative physical outcomes^(3)^. Other studies have also reported negative outcomes associated with energy drink consumption, including similar adverse events listed above as well as increased cardiac arrythmia, lower levels of sleep, increased stress, anxiety, anger, hepatic alterations, and other detrimental mental and physical effects^(5–11)^. Additionally, some users mix energy drinks with alcohol, which can lead to further risky behaviours leading to adverse outcomes^(12–15)^. Energy drink has been found to be associated with other risky behaviours including drug use, poor dietary habits, sensation seeking and worse school performance^(16)^. Finally, studies have tended to focus on either younger consumers^(16–18)^ or members of the military^(19–21)^. Given these concerns, and relatively little data among non-military adults, we comprehensively assessed demographic and behavioural factors related to energy drink consumption, particularly high-risk behaviours such as alcohol and drug use, among participants in two large cohorts in the USA.

## Methods

### Study population

**Fig. 1:**
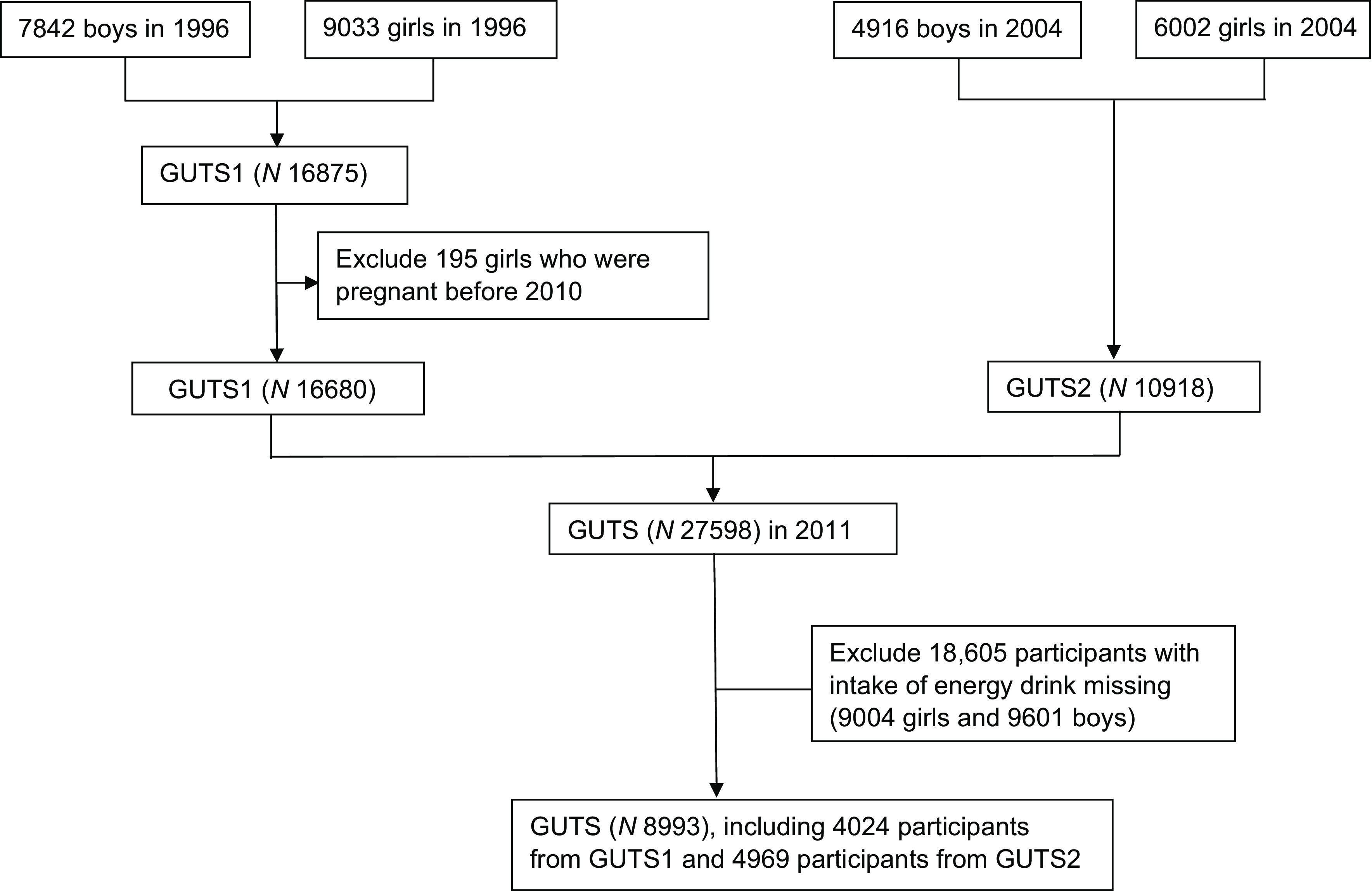
A flow diagram of sample selection in the Growing Up Today Study (GUTS)

**Fig. 2:**
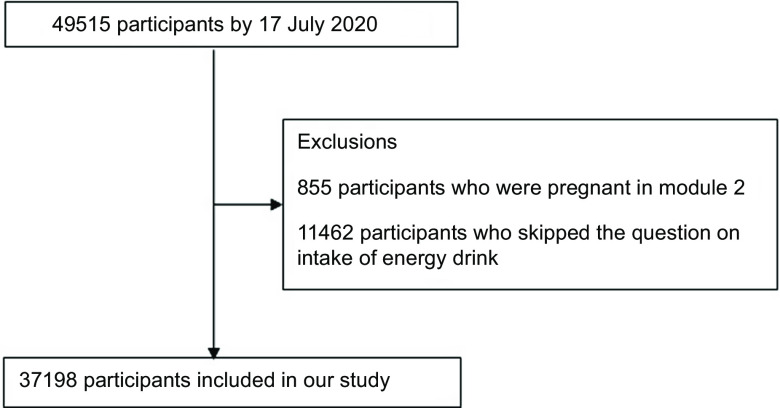
Flow diagram of sample selection in the Nurses’ Health Study 3 (NHS3)

We conducted cross-sectional analysis using data from the Nurses’ Health Study 3 (NHS3) and Growing Up Today Study (GUTS)^(22)^. The NHS3 is an open, web-based prospective cohort study of female nurses and nursing students in North America. Enrolment started in 2010 and is ongoing. Participants complete a baseline questionnaire and follow-up questionnaires approximately every 6 months. As of 17 July 2020, 49 515 female participants have joined the study. GUTS is an ongoing prospective cohort study of young adults. The cohort was established in 1996 with the recruitment of 16 875 children, age 9–15 years (GUTS1) and expanded in 2004 with the enrolment of an additional 10 918 children aged 9–15 years (GUTS2). Participants complete follow-up questionnaires every 1–3 years. Because of age differences between the two enrolment cycles, these two groups were followed separately until 2013, when all participants were adults, and a common follow-up schedule and follow-up materials were used. For this study, we included NHS3 participants (age 20–55 years) who completed a diet assessment in the first follow-up questionnaire and GUTS participants (age 16–31 years) who completed a diet assessment in 2011, when intake of energy drinks was first assessed in this cohort. Eighty per cent of participants completed the follow-up questionnaire between 2010 and 2013. After excluding female participants who skipped the question on intake of energy drink and were pregnant at the time of diet assessment (excluded 9004) and males who did not skip the question about energy drinks (excluded 9601), the study included 37 198 participants from NHS3 and 8993 participants from GUTS. We combined the data from both cohorts for our analysis, in part, due to low overall levels of energy drink consumption, as described further in the discussion section. Participant selection flowcharts for GUTS and NHS3 are shown in Fig. 1 and 2, respectively.

### Assessment of energy drink use

Diet was assessed using an extensively validated FFQ^(23,24)^. Participants reported how often, on average, they consumed 131 foods and beverages listed in the questionnaire during the past year. Participants were asked to report their intake of ‘Energy drinks (e.g. Red Bull, Rock Star and Monster), 1 can’ in one of nine categories of increasing frequency of intake ranging from never or less than once per month to six or more times per d. For the purpose of this study, energy drink users were defined as those who reported consuming energy drinks at least once per month. The selected frequency category for each food item was then converted to daily intake. Food intake under each category was then combined to obtain the total intake of fruits, vegetables, fish and shellfish, red meat, sugar-sweetened beverages, etc. The FFQ used here has been previously shown to be of high validity^(23,24)^ Finally, use of energy drinks was assessed in the second questionnaire in NHS3 and in 2011 in GUTS.

### Assessment of demographic and behavioural data

Sociodemographic and behavioural data were obtained from responses to follow-up questionnaires completed closest to the time of energy drink consumption assessment when available, and otherwise from each participant’s demographic information collected at baseline (online Supplementary Table 1). Participants reported their race and ethnicity using categories defined by the US Census Bureau (Non-Hispanic White, Non-Hispanic Black, Hispanic, Asian and Other). Other demographic characteristics reported by participants included highest level of education achieved (< Bachelor’s, Bachelor’s, ≥ Master’s degree), geographical region of residence (Northeast, Midwest, West and South), marital status (never married, married, divorced, separated, widowed and domestic partnership) and sexual orientation (completely heterosexual, mostly heterosexual, bisexual, mostly homosexual, completely homosexual and not sure).

We decided to assess behaviours that could be harmful to human health based on findings from existing literature such as drug/alcohol use^(15–18,25–27)^, as well as other potentially risky behaviours identified by the authors that were already collected as part of the GUTS/NHS 3 questionnaires. Data on health-related behaviours and behavioural risk factors included smoking status (current or past smoker), e-cigarette use (yes/no), usual duration of overnight sleep (< 7, 7–9, > 9 h specifically, we asked about hours of sleep on work-free days without obligations in NHS3), tanning bed use (yes/no), use of birth control among female participants (Depo Provera, Other hormonal contraception, Vasectomy, Tubal Ligation, Oral Contraceptive, Foam/Jelly/Sponge, Diaphragm/Cervical Cap, Condom, Rhythm/Natural Family Planning, Intrauterine Device and yes/no), preventive physical exam/doctor’s visit in the past 2 years (yes/no), lifetime number of sexual contacts/partners, marijuana use (yes/no), use of illegal drugs besides marijuana (cocaine, heroin, ecstasy, lysergic acid diethylamide (LSD/acid)), mushrooms (shrooms) or any other hallucinogen, crystal meth (methamphetamine, crank, tweak), other amphetamines (uppers, speed)), binge drinking (yes/no), use of multivitamin supplements (yes/no), and use of muscle enhancing products (yes/no, protein powder or shake, creatine, weight loss shakes/drinks, amino acids, hydroxymethylbutyrate /HMB, dehydroepiandrosterone/DHEA, growth hormone, anabolic/injectable steroids). The most recent self-reports of height and weight, which have been previously found to be validly reported in GUTS and other cohorts of nurses^(28)^, were used to calculate the BMI as weight (kg) divided by height squared (m^2^) and then grouped into WHO reference categories (< 25 kg/m^2^, 25–30 kg/m^2^, ≥ 30 kg/m^2^).

Participants’ physical activity was assessed by means of self-report questionnaires in 2015 in GUTS and the second questionnaire in NHS3. Participants were asked about the amount of time that they spent per week, on average, in each of the following physical activities: walking; jogging; running; bicycling; playing tennis; other aerobic exercise (aerobic, dance, ski or stair machine, etc.), lower intensity exercise (yoga, stretching and toning) and other vigorous activities (e.g. lawn mowing). The amount of total reported physical activity was calculated as energy expenditure in hours per week.

A single diet score (based on criteria of the *American Heart Association* (AHA)) was defined using *a priori* cut-offs^(29)^, looking at consumption quantities for various foods, including: fruits/vegetables, fish and shellfish, Na, sugar-sweetened beverages (SSB), and whole grains, as well as nuts/legumes/seeds, processed meat, and saturated fat. For healthy foods, such as fruits and vegetables or fish, participants received a score ranging from 0 to 10 points based on consumption level – where a score of 0 indicated they did not consume the food at all and 10 indicated that they consumed the optimal level of said food according to the AHA. For unhealthy foods (e.g. processed meats, SSB), a score of 0 indicated intake higher than that of the 80–90th percentile among US adults, while a score of 10 meant that the food was consumed at or below the target intake level^(29)^.

### Statistical analysis

We conducted both basic descriptive and quantitative analyses to characterise demographic and behavioural factors in relation to energy drink consumption. Descriptive analysis addressed the distributions of demographic and behavioural factors by reported energy drink use among each cohort. Univariate/multivariable logistic models were used for bivariate analyses. We examined associations between demographic and behavioural factors and the odds of energy drink consumption using logistic regression. We applied both univariable and multivariable logistic regression models. As to multivariable analysis, we simultaneously included all demographic and behavioural factors into the regression model and used missing indicators for variables with missing values. As for pooled analysis, we combined individual data in NHS3 and GUTS into one dataset and conducted regression analyses in the pooled dataset. We tested for heterogeneity in associations between cohorts by including interaction terms of each covariate and cohort into the model, and P_for interaction_ was obtained using likelihood ratio test comparing models with and without interaction terms. Given the multiple demographic and behavioural factors considered, we corrected for multiple testing using Bonferroni correction and considered results significant or significant heterogeneity in associations across cohorts with *P* value<0·05/37 (=0·001), where 37 was the number of tests performed.

Missing data for all variables were presented in Supplementary table 2. Given that we included all variables simultaneously into the model for multivariable regression, we used missing indicators for variables with missing values to avoid issues of model convergence. In sensitivity analysis, for variables that have skewed distributions, we imputed missing values as the value that has the highest percentage, i.e. ‘no’ for smoking status, use of tanning bed, binge drinking, marijuana use, and multivitamin use, married/partner for marriage status, and completely heterosexual for sexual orientation. We fitted multivariable logistic regression including those imputed variables to obtain OR of these variables with energy drink intake and then added the rest of the variables into the multivariable model one by one to obtain OR of that variable. All statistical tests were two-sided and performed using SAS version 9.2 for UNIX (SAS Institute Inc).

## Results

Our study included 46 390 individuals, with 9088 (19·5 %) participants from the GUTS and 37 302 (80·5 %) were from the NHS3. The overall levels of energy drink consumption are shown in Table 1. 40 269 participants (86·8 %) reported that they consumed fewer than one energy drink product per month, while 3540 (7·6 %) reported consuming 1–3 energy drinks per month and 2581 (5·6 %) reported consuming more than three energy drinks per month. Of those 2581 individuals, 8·4 % (0·5 % of the total study sample) reported daily consumption of energy drinks and 3·9 % (0·2 % of the total sample) reported consuming more than one energy drink per d.

**Table 1:** Distribution of intakes of energy drink in Nurses’ Health Study 3 (NHS3) and Growing Up Today Study (GUTS)

Energy drinks	NHS3 (*n* 37 302)	%	GUTS (*n* 8993)	%	Total *(n* 46 295)	%
Never, or less than once per month	33 248	89·1	6936	77·1	40 184	86·8
1–3 per month	2411	6·5	1122	12·5	3533	7·6
1 per week	696	1·9	484	5·4	1180	2·5
2–4 per week	602	1·6	289	3·2	891	1·9
5–6 per week	113	0·3	79	0·9	192	0·4
1 per d	186	0·5	29	0·3	215	0·5
> 1 per d	46	0·1	54	0·6	100	0·2

Table 2 shows available demographic and risk factor data for comparison by energy drink consumer status across the cohorts. Over 75 % of individuals reported having at least a bachelor’s degree, with 8405 (22·6 % of those reporting) indicating a master’s degree or higher education. Energy drink consumption was less likely among higher education levels compared to the lowest ones. Most participants (about 90 %) self-identified as Caucasian across the cohorts. Most participants were non-smokers, with the lowest rates of smoking seen in GUTS2; smokers were more likely to be energy drink consumers. Less than 1 % of NHS3 participants reported e-cigarette use, but data on e-cigarette use were not collected in the other cohorts. E-cigarette use was also more common among those who reported energy drink use. GUTS1 and GUTS2 participants were more likely than NHS3 members to report binge drinking, defined as four or more alcoholic drinks over a few hours. More than half (51·5 %) of GUTS1 participants reported marijuana use, exceeding use indicated by data from the two other cohorts. GUTS1 participants also were more likely to report use of illegal drugs (20·0 %) compared to NHS3 participants (13·3 %); GUTS2 did not collect data on this exposure.

**Table 2:** Distribution of exposure according to intake of energy drinks in Growing Up Today Study (GUTS) and Nurses’ Health Study 3 (NHS3)

			GUTS						NHS3			
	Non-consumers (*n* 6936)	%	Consumers (*n* 2057)	%	Overall (*n* 8993)	%	Non-consumers (*n* 33 248)	%	Consumers (*n* 4054)	%	Overall (*n* 37 302)	%
Demographic and geographical												
Age												
< 25 years	4185	60·3	1472	71·6	5657	62·9	3905	10·7	84	13·4	3989	10·7
25–30 years	2734	39·4	581	28·3	3315	36·9	9249	25·3	171	27·2	9420	25·3
30–35 years	17	0·3	4	0·2	21	0·2	7625	20·9	109	17·3	7734	20·8
> 35 years	0	0·0	0	0·0	0	0·0	15 790	43·2	265	42·1	16 055	43·2
Sex												
Female	4835	69·7	1001	48·7	5836	64·9	33 248	89·1	4054	10·9	37 302	100
Male	2101	30·3	1056	51·3	3157	35·1	0	0·0	0	0·0	0	0·0
Education												
Without a bachelor	1221	22·5	362	26·8	1583	23·4	22 210	68·6	368	77·3	22 578	68·7
Bachelor	2646	48·8	735	54·3	3381	49·9	7577	23·4	78	16·4	7655	23·3
Master or higher	1559	28·7	256	18·9	1815	26·8	2586	8·0	30	6·3	2616	8·0
Race												
Non-Hispanic White	6646	95·8	1966	95·6	8612	95·8	34 124	93·3	567	90·1	34 691	93·3
Black	12	0·2	6	0·3	18	0·2	810	2·2	27	4·3	837	2·3
Hispanic	94	1·4	36	1·8	130	1·5	364	1·0	10	1·6	374	1·0
Asian	79	1·1	13	0·6	92	1·0	869	2·4	15	2·4	884	2·4
Other	105	1·5	36	1·8	141	1·6	402	1·1	10	1·6	412	1·1
Sexual orientation												
Completely heterosexual	6584	95·8	1958	95·9	8542	95·8	24 472	96·2	218	91·2	24 690	96·1
Bisexual/homosexual	288	4·2	84	4·1	372	4·2	971	3·8	21	8·8	992	3·9
Marriage												
Never married	5375	78·2	1736	85·4	7111	79·8	1416	35·5	99	45·6	1515	36·0
Married/partner	1458	21·2	283	13·9	1741	19·5	2277	57·0	99	45·6	2376	56·5
Divorced/separated/widowed	43	0·6	14	0·7	57	0·6	299	7·5	19	8·8	318	7·6
Region												
Northeast	2180	31·5	603	29·3	2783	31·0	7344	23·4	64	18·7	7408	23·4
Midwest	2330	33·6	729	35·5	3059	34·1	8757	27·9	97	28·4	8854	27·9
South	1184	17·1	358	17·4	1542	17·2	8448	26·9	102	29·8	8550	27·0
West	1234	17·8	365	17·8	1599	17·8	6810	21·7	79	23·1	6889	21·7
Lifestyle												
Smoking												
Never smokers	4213	87·5	984	72·8	5197	84·3	5572	79·9	87	64·0	5659	79·6
Current or past smokers	603	12·5	368	27·2	971	15·7	1401	20·1	49	36·0	1450	20·4
BMI												
< 25 kg/m^2^	4885	72·5	1308	65·2	6193	70·8	19 616	54·0	240	38·6	19 856	53·8
25–30 kg/m^2^	1277	19·0	483	24·1	1760	20·1	8541	23·5	169	27·2	8710	23·6
≥ 30 kg/m^2^	578	8·6	216	10·8	794	9·1	8155	22·5	213	34·2	8368	22·7
Sleep												
7–9 h	4866	77·9	1241	71·7	6107	76·6	775	19·1	77	30·4	852	19·7
< 7 h	1290	20·7	469	27·1	1759	22·1	208	5·1	20	7·9	228	5·3
> 9 h	91	1·5	21	1·2	112	1·4	3082	75·8	156	61·7	3238	75·0
Physical activity												
Low	1849	32·2	400	27·3	2249	31·2	11 736	33·4	104	28·3	11 840	33·3
Medium	2150	37·5	548	37·4	2698	37·5	11 743	33·4	102	27·7	11 845	33·3
High	1736	30·3	517	35·3	2253	31·3	11 679	33·2	162	44·0	11 841	33·3
Diet quality												
Low	1909	30·3	728	41·7	2637	32·8	10 912	31·8	67	37·6	10 979	31·9
Medium	2148	34·1	541	31·0	2689	33·4	11 671	34·0	54	30·3	11 725	34·0
High	2246	35·6	477	27·3	2723	33·8	11 702	34·1	57	32·0	11 759	34·1
Supplements for muscle enhancement												
No	1647	78·4	795	75·3	2442	77·4	2377	78·4	60	64·5	2437	78·0
Yes	454	21·6	261	24·7	715	22·7	655	21·6	33	35·5	688	22·0
Physical exam in the past 1–2 years												
Yes	5804	84·0	1673	81·8	7477	83·5	25 834	70·6	334	53·1	26 168	70·4
No	1103	16·0	373	18·2	1476	16·5	10 735	29·4	295	46·9	11 030	29·7
Birth control, female participants only												
No	1378	33·2	216	26·9	1594	32·2	6201	31·2	100	27·3	6301	31·1
Yes	2776	66·8	587	73·1	3363	67·8	13 675	68·8	266	72·7	13 941	68·9
Multivitamin supplementation												
No	3652	59·9	1069	62·2	4721	60·4	36 098	98·7	523	83·2	36 621	98·5
Yes	2448	40·1	649	37·8	3097	39·6	471	1·3	106	16·9	577	1·6
Risky behaviours												
E-cigarette use												
No	5749	97·6	1388	90·8	7137	96·2	1000	89·1	115	65·7	1115	86·0
Yes	143	2·4	141	9·2	284	3·8	122	10·9	60	34·3	182	14·0
Tanning bed												
No	5811	84·2	1516	74·4	7327	81·9	2352	60·2	126	67·4	2478	60·5
Yes	1094	15·8	522	25·6	1616	18·1	1556	39·8	61	32·6	1617	39·5
Binge drinking												
No	1714	29·7	197	12·5	1911	26·0	10 353	45·7	45	20·5	10 398	45·4
Yes	4060	70·3	1377	87·5	5437	74·0	12 318	54·3	175	79·6	12 493	54·6
Marijuana use												
No	3865	64·1	761	47·8	4626	60·7	12 149	48·3	101	43·4	12 250	48·3
Yes	2162	35·9	832	52·2	2994	39·3	12 998	51·7	132	56·7	13 130	51·7
Use of illegal drugs												
No	2746	82·9	459	64·4	3205	79·7	31 674	86·6	554	88·1	32 228	86·6
Yes	565	17·1	254	35·6	819	20·4	4895	13·4	75	11·9	4970	13·4
Number of persons with sexual contact												
1 person	270	25·9	59	14·8	329	22·8	4620	19·4	15	6·8	4635	19·3
2 persons	190	18·2	48	12·0	238	16·5	2262	9·5	13	5·9	2275	9·5
3–5 persons	304	29·2	109	27·3	413	28·6	6041	25·4	32	14·5	6073	25·3
> 5 persons	279	26·8	183	45·9	462	32·0	10 867	45·7	161	72·9	11 028	45·9

We present the associations of demographic and behavioural factors with odds of being an energy drink consumer in Table 3. For GUTS-only participants, being female, and for both GUTS and NHS3 participants, higher education levels, were consistently associated with lower odds of energy drink consumption in multivariable logistic regressions (Table 3). Black study participants were more likely to consume energy drinks compared to Caucasian participants (pooled OR (pOR: 2·10. 95 % CI: 1·41, 3·14)). Marital status was not associated with energy drink consumption. Those self-identifying with bisexual/homosexual orientation were statistically significantly more likely to consume energy drinks based on results from the NHS3 univariable analyses (OR: 2·43, 95 % CI: 1·54, 3·82), but sexual orientation was not significantly associated with energy drink intake in the other cohort or pooled analyses.

**Table 3:** OR of consuming energy drinks characteristics related to intake of energy drinks in Growing Up Today Study (GUTS) and Nurses’ Health Study 3 (NHS3)

	GUTS				NHS3				Pooled			
	Univariable OR	95 % CI	Multivariable-adjusted OR	95 % CI	Univariable OR	95 % CI	Multivariable-adjusted OR	95 % CI	Multivariable-adjusted OR	95 % CI	*P* value	*P* for heterogeneity
Demographic and geographical												
Age												0·83
< 25 years	Ref		Ref		Ref		Ref		Ref			
25–30 years	0·93	0·75, 1·15	0·89	0·70, 1·12	0·86	0·66, 1·12	0·96	0·70, 1·31	0·94	0·78, 1·13	0·50	
30–35 years	1·03	0·34, 3·11	0·73	0·20, 2·61	0·66	0·50, 0·89	0·60	0·43, 0·86	0·65	0·49, 0·86	0·002	
> 35 years	NA		NA		0·78	0·61, 1·00	0·50	0·36, 0·70	0·60	0·47, 0·75	<0·001	
Sex												
Female	Ref		Ref						Ref			
Male	2·34	2·11, 2·59	2·31	1·84, 2·89	NA		NA		3·15	2·24, 4·43	<0·001	
Education												0·002
Without a bachelor	Ref		Ref		Ref		Ref		Ref			
Bachelor	1·04	0·89, 1·20	1·04	0·89, 1·23	0·62	0·49, 0·79	0·66	0·51, 0·86	0·91	0·80, 1·03	0·13	
Master or higher	0·69	0·57, 0·83	0·78	0·63, 0·96	0·70	0·48, 1·02	1·00	0·66, 1·51	0·74	0·62, 0·89	0·001	
Race												0·49
Non-Hispanic White	Ref		Ref		Ref		Ref		Ref			
Black	2·25	0·84, 6·02	2·93	0·93, 9·17	2·01	1·36, 2·97	2·10	1·33, 3·32	2·10	1·41, 3·14	<0·001	
Hispanic	1·15	0·78, 1·70	1·25	0·83, 1·90	1·65	0·88, 3·12	1·84	0·90, 3·75	1·29	0·90, 1·86	0·17	
Asian	0·60	0·33, 1·08	0·80	0·42, 1·51	1·04	0·62, 1·74	0·97	0·55, 1·71	0·86	0·56, 1·30	0·47	
Other	1·10	0·75, 1·61	0·99	0·65, 1·50	1·50	0·80, 2·82	1·52	0·75, 3·11	1·14	0·79, 1·64	0·50	
Sexual orientation												0·02
Completely heterosexual	Ref		Ref		Ref		Ref		Ref			
Bisexual/homosexual	1·02	0·79, 1·31	0·80	0·61, 1·05	2·43	1·54, 3·82	1·29	0·76, 2·18	0·91	0·71, 1·16	0·43	
Marriage												0·53
Never married	Ref		Ref		Ref		Ref		Ref			
Married/partner	0·76	0·66, 0·88	0·91	0·77, 1·08	0·62	0·47, 0·83	0·80	0·57, 1·14	0·94	0·81, 1·09	0·42	
Divorced/separated/widowed	1·30	0·70, 2·39	0·91	0·47, 1·78	0·91	0·55, 1·51	1·07	0·60, 1·92	1·10	0·72, 1·68	0·68	
Region												0·54
Northeast	Ref		Ref		Ref		Ref		Ref			
Midwest	1·11	0·98, 1·26	1·10	0·97, 1·26	1·27	0·93, 1·75	1·40	1·00, 1·98	1·16	1·02, 1·31	0·02	
South	1·12	0·96, 1·30	1·14	0·97, 1·34	1·39	1·01, 1·90	1·55	1·10, 2·17	1·20	1·04, 1·38	0·02	
West	1·08	0·93, 1·26	1·14	0·97, 1·34	1·33	0·96, 1·85	1·64	1·14, 2·35	1·21	1·04, 1·40	0·01	
Lifestyle												
Smoking												0·84
Never smokers	Ref		Ref		Ref		Ref		Ref			
Current or past smokers	3·40	2·91, 3·97	1·88	1·57, 2·26	2·24	1·57, 3·20	2·24	1·51, 3·33	1·97	1·68, 2·31	<0·001	
BMI												0·12
< 25 kg/m^2^	Ref		Ref		Ref		Ref		Ref			
25–30 kg/m^2^	1·49	1·32, 1·68	1·28	1·12, 1·46	1·62	1·33, 1·97	1·36	1·09, 1·69	1·30	1·16, 1·45	<0·001	
≥ 30 kg/m^2^	1·55	1·30, 1·83	1·48	1·23, 1·78	2·14	1·77, 2·57	1·86	1·50, 2·32	1·64	1·43, 1·88	<0·001	
Sleep												0·01
7–9 h	Ref		Ref		Ref		Ref		Ref			
< 7 h	1·39	1·23, 1·58	1·33	1·16, 1·52	0·97	0·58, 1·62	0·94	0·52, 1·70	1·28	1·12, 1·45	<0·001	
> 9 h	0·89	0·55, 1·43	0·75	0·45, 1·26	0·51	0·38, 0·68	0·68	0·49, 0·95	0·63	0·50, 0·81	<0·001	
Physical activity												0·10
Low	Ref		Ref		Ref		Ref		Ref			
Medium	1·14	0·99, 1·32	1·10	0·94, 1·28	0·98	0·75, 1·29	1·12	0·84, 1·51	1·09	0·95, 1·25	0·20	
High	1·29	1·11, 1·50	1·12	0·95, 1·31	1·57	1·22, 2·01	1·50	1·14, 1·98	1·22	1·06, 1·40	0·005	
Diet quality												<0·001
Low	Ref		Ref		Ref		Ref		Ref			
Medium	0·66	0·58, 0·75	0·77	0·67, 0·88	0·75	0·53, 1·08	0·78	0·54, 1·11	0·77	0·67, 0·87	<0·001	
High	0·59	0·51, 0·67	0·72	0·63, 0·84	0·79	0·56, 1·13	0·80	0·56, 1·14	0·73	0·64, 0·83	<0·001	
Supplements for muscle enhancement												0·06
No	Ref		Ref		Ref		Ref		Ref			
Yes	1·20	1·00, 1·42	1·30	1·07, 1·59	2·00	1·29, 3·08	1·95	1·19, 3·19	1·39	1·16, 1·67	<0·001	
Physical exam in the past 1–2 years												0·09
Yes	Ref		Ref		Ref		Ref		Ref			
No	1·28	1·12, 1·46	1·09	0·94, 1·26	2·13	1·82, 2·49	0·87	0·71, 1·07	1·02	0·91, 1·15	0·75	
Birth control, female participants only												0·62
No	Ref		Ref		Ref		Ref		Ref			
Yes	1·53	1·29, 1·82	1·26	1·04, 1·51	1·21	0·96, 1·52	1·37	1·06, 1·77	1·29	1·11, 1·49	<0·001	
Multivitamin supplementation												0·03
No	Ref		Ref		Ref		Ref		Ref			
Yes	0·91	0·82, 1·02	1·01	0·90, 1·14	15·53	12·37, 19·51	1·46	1·09, 1·94	1·07	0·96, 1·19	0·24	
Risky behaviours												
E-cigarette use												0·06
No	Ref		Ref		Ref		Ref		Ref			
Yes	4·08	3·21, 5·20	2·07	1·58, 2·70	4·28	2·97, 6·16	3·20	2·15, 4·75	2·44	1·96, 3·04	<0·001	
Tanning bed												<0·001
No	Ref		Ref		Ref		Ref		Ref			
Yes	1·85	1·64, 2·08	2·37	2·07, 2·72	0·73	0·54, 1·00	0·60	0·42, 0·85	1·95	1·71, 2·21	<0·001	
Binge drinking												0·49
No	Ref		Ref		Ref		Ref		Ref			
Yes	3·38	2·87, 3·97	2·38	1·99, 2·84	3·27	2·35, 4·54	2·97	2·08, 4·24	2·66	2·27, 3·11	<0·001	
Marijuana use												<0·001
No	Ref		Ref		Ref		Ref		Ref			
Yes	2·47	2·19, 2·78	1·53	1·32, 1·76	1·22	0·94, 1·59	0·55	0·39, 0·78	1·29	1·13, 1·47	<0·001	
Use of illegal drugs												0·59
No	Ref		Ref		Ref		Ref		Ref			
Yes	2·69	2·25, 3·21	1·27	1·02, 1·57	0·88	0·69, 1·12	1·63	1·14, 2·32	1·29	1·08, 1·55	0·004	
Number of persons with sexual contact												0·001
1 person	Ref		Ref		Ref		Ref		Ref			
2 persons	1·16	0·76, 1·77	0·97	0·62, 1·52	1·77	0·84, 3·72	1·92	0·89, 4·14	1·14	0·78, 1·67	0·51	
3–5 persons	1·64	1·15, 2·34	1·20	0·82, 1·75	1·63	0·88, 3·01	1·59	0·84, 3·03	1·23	0·90, 1·70	0·20	
> 5 persons	3·00	2·14, 4·21	1·44	1·00, 2·08	4·56	2·68, 7·75	3·70	2·08, 6·56	1·87	1·39, 2·51	<0·001	

Univariate analysis used a logistic model.

Multivariate analysis used a logistic model that simultaneously included all variables in the model.

Pooled analysis combined individual data from NHS3 and GUTs into a single dataset and conducted regression analysis in the pooled dataset. We tested for heterogeneity in association between cohorts by including interaction terms of each covariate and cohort into the model, and *P*_*for interaction*_ was obtained using likelihood ratio test comparing models with and without interaction terms.

Several behavioural factors were also associated with energy drink consumption as seen in Table 3. Smokers were more likely within and across cohorts to consume energy drinks than non-smokers (pOR: 1·97. 95 % CI: 1·68, 2·31). Higher BMI levels (25–30 kg/m^2^ and ≥ 30 kg/m^2^ compared to < 25 kg/m^2^) were, overall, also associated with consumption; those with lower, healthier BMI levels were less likely to consume energy drinks. The pooled model showed that those with high-quality diets were statistically significantly less likely to consume energy drinks compared to those with lower-quality diets; however, the individual cohort models revealed that high-quality diet was not associated with energy drink consumption in the NHS3. Similarly, results for the relationship between physical activity and energy drink intake varied across and within cohorts, including statistically significant associations between high physical activity and energy drink consumption. Additionally, individuals who reported that they used supplements for muscle enhancement were statistically significantly more likely to consume energy drinks based on the GUTS1 age- and sex-adjusted and multivariable models, as well as the pooled multivariable model, but the relationship between muscle enhancement supplements and energy drink intake was not statistically significant in either of the NHS3 models. E-cigarette use (as-aOR: 2·44, 95 % CI: 1·96, 3·04), tanning bed use (pOR: 1·87, 95 % CI: 1·73, 2·24), binge drinking (pOR: 2·66, 95 % CI: 2·27, 3·11), marijuana use (pOR: 1·29, 95 % CI: 1·13, 1·47) and illegal drug use (pOR: 1·29, 95 % CI: 1·08, 1·55) were all strongly significantly associated with energy drink consumption in the pooled analysis.

Finally, we examined potential associations of all variables with energy drink intake, accounting for missing values. As shown in Supplementary Table 3, the findings are nearly identical to those in the main analysis. Male participants were more likely to consume energy drinks (aOR: 2·86, 95 % CI: 2·56, 3·21), as were Black participants (pOR: 2·08, 95 % CI: 1·44, 3·01). Higher BMI were also associated with energy drink intake (for both higher BMI categories compared to < 25 kg/m^2^), as were e-cigarette use (pOR: 4·56, 95 % CI: 3·63, 5·74), tanning bed use (pOR: 2·37, 95 % CI: 2·11, 2·67), binge drinking (pOR: 1·23, 95 % CI: 1·11, 1·36) and illegal drug use (pOR: 1·49, 95 % CI: 1·25, 1·77).

## Discussion

This study shows that energy drink intake is associated with risky behaviours and suboptimal physical status, such as obesity, e-cigarette use, smoking, binge drinking, and use of illicit drugs, and highlights the complexity of the public health problems related to consumption of these products. Our results are also consistent with previously reported associations between energy drink intake and risky behaviours, especially among teenagers and young adults, including illicit drug use, alcohol dependence and binge drinking, prescription drug misuse, and other harmful outcomes^(16,17,25,26,30)^. For example, Buja et al. (2017)^(31)^ found that caffeinated energy drink consumption was associated with negative impacts on digital well-being, revealed by problematic social network site usage among young teenagers in North-eastern Italy. An Icelandic study additionally showed that adolescent caffeine consumption was associated with self-reported physically aggressive behaviours towards others, including punching and kicking other individuals^(32)^. Finally, researchers have also reported significant associations between increased energy drink consumption and suicide attempts among teenagers in South Korea and Ontario, Canada^(7,33)^. Overall, energy drink consumption has been correlated with risky behaviours that can have short-term, long-term, and/or permanent negative effects, especially among youth.

Much of the current literature on caffeinated energy drinks focuses on teenagers and younger/college-age adults^(17,18)^, as well as military personnel who may be of a similar age as many enlist or are conscripted as young adults^(19–21)^. Few studies, however, appear to assess the effects of caffeinated energy drinks among adults who are older than college age, and even fewer look together at risky behaviours among them and their offspring. Lieberman et al. analysed 2007–2012 NHANES data for adults aged 19+ years, primarily looking at demographic factors, as well as some health status characteristics and risky behaviours associated with all caffeine intake (not just caffeinated energy drink consumption); they found that smoking, higher calorie intake and alcohol consumption (when adjusted for employment factors) were associated with increased caffeine usage^(34)^. In a study of energy drink intake among illicit drug users who participated in the online 2014 Global Drug Survey of individuals aged 16 years or older, Peacock et al. (2017)^(27)^ found that over two-thirds of participants reported ever consuming energy drinks and higher levels of energy drink use were associated with several risky health behaviours, including use of tobacco, marijuana, amphetamine, 3,4-methylenedioxymethamphetamine (MDMA/ ecstasy) and cocaine.

Given the general dearth of energy drink consumption and risky behaviour data among adults, and the potential for adverse health outcomes from these behaviours, we believe that our study provides a unique opportunity to assess the behaviours that may accompany energy drink consumption among slightly older adults – study participants were generally older than those considered in much of the literature, with participant mean baseline ages ranging from a college student age common in the literature of 19·61 years (GUTS non-consumers) to a much older 33·96 years (NHS3 non-consumers). Furthermore, our study found several risky behaviours associated with energy drink consumption in a large, robust sample size. Additionally, we assessed certain risky behaviours related to energy drink use that have not often been assessed in the literature, including tanning bed use, lifetime number of sexual partners and use of muscle enhancement supplements. These findings help shed light on health issues and activities of concern to both the general public and, perhaps, also serve as a warning sign for medical and other health practitioners to look for other unsafe behaviours as well.

A potential limitation with our study is that the two cohorts are fundamentally very different in terms of their sample source. However, we believe that the benefits of pooling the cohorts outweigh this limitation. First, although population characteristics are different between the two cohorts, we found similar associations for many variables, showing the robustness of our findings. Second, the heterogeneity in associations for some variables allows us to have a better understanding of the associated patterns of energy drink intake. Third, by combining the NHS3 and GUTS, our study has ample power to achieve a strong conclusion of our research question, particularly in the presence of overall low energy drink consumption. Another limitation is that some of the data used for the GUTS analysis is from 2011, meaning that behaviours and other relevant information may have changed since the data were collected.

Limitations of addressing different study periods and exposure windows notwithstanding, as previously mentioned, an analysis of CAERS and NPDS data found that consumption of caffeinated energy drinks could lead to a variety of serious and life-threatening events, including tachycardia and other cardiac disorders, nervous system irregularities, nausea/vomiting and even death^(3)^. A recent systematic review of thirty-two studies found that insomnia was frequently associated with energy drink consumption, both among children and adults^(35)^. Other studies have also reported associations between energy drink consumption and cardiac outcomes, including life-threatening arrhythmias^(10,11)^, and possibly hepatic and kidney damage^(36)^. While most consumption of caffeine can be generally considered safe^(37)^, use of caffeinated energy drinks can present health-threatening risks^(3)^ that warrant consideration in conjunction with the other unhealthy behaviours addressed in this analysis.

Certain limitations constrained this study, which nevertheless identified several characteristics and risky behaviours correlated with energy drink use in adults. First, even though the study used data from longitudinal cohort studies, the cross-sectional analysis restricted our ability to assess temporality between consumption of energy drinks and behaviours^(17)^; therefore, we are unable to determine if consumption of energy drinks actually led to the risky behaviours, if the risky behaviours simply happened concurrently with energy drink use, and/or if the behaviours actually preceded energy drink intake. Furthermore, we are unable to see if and/or how energy drink consumption may have changed over time and whether such variability would affect the associations detected in the cross-sectional analysis. When data from future iterations of questionnaires among the same cohorts become available, conducting longitudinal analysis will be possible, which in turn will help further elucidate these potential relationships and provide better insight to potentially inform consumer outreach and education and public health interventions going forward. Finally, we could not analyse the relationship between the specific products (e.g. individual brands) and the behaviours assessed, because user characteristics and health outcomes can vary by product^(3)^.

## Conclusion

Findings from this study indicate that energy drink consumption is associated with high BMI (25–30 kg/m^2^ and ≥ 30 kg/m^2^) and poor diet quality. Furthermore, findings from this study suggest that any potential health problems due to high BMI and poor diet could be further impacted by several risky behaviours, including smoking and/or e-cigarette use, binge drinking, and illegal drug use that are associated with use of energy drinks, leading to increased risk of morbidity and mortality. Our findings are important to help public health practitioners develop population-level targeted and effective interventions, including education and outreach, to reduce and prevent adverse health outcomes stemming from risky behaviours related to use of energy drinks. Finally, clinicians should be made aware of these relationships to better identify potentially vulnerable patients to provide specific health advice and care.

## Supplementary material

For supplementary material accompanying this paper visit https://doi.org/10.1017/S1368980022001902. click here to view supplementary material
