# Supplementary material for: Demographic and behavioural correlates of energy drink consumption
Source: Public Health Nutr. 2022 Oct 10;26(7):1424–35. doi: 10.1017/S1368980022001902 (PMC10258655; doi:10.1017/S1368980022001902)
Supplement: Supplementary file 1 [file S1368980022001902sup001.docx]

**Supplemental** **Table 1. Time of Assessment of Demographic and Behavioral Data.**

| **Variable** | **Year Guts 1** | **Year Guts 2** | **NHS 3*** |
| --- | --- | --- | --- |
| **Sex** | 1996 | 2004 | Mod 1 |
| **Race** | 1996 | 2004 | Mod 1 |
| **Age** | 2010 | 2011 | Mod 2 |
| **Education** | 2014 | 2014 | Mod 1 |
| **Sexual Orientation** | 2010 | 2011 | Mod 5 |
| **Marital Status** | 2010 | 2011 | Mod 1 |
| **Geographic Region** | 2010 | 2011 | Mod 1 |
| **Smoking** | 2010 | 2011 | Mod 1 |
| **Obesity** | 2010 | 2011 | Mod 1 |
| **Sleep** | 2008 | 2010 | Mod 1 |
| **Physical Activity** | 2015 | 2015 | Mod 2 |
| **Diet** | 2011 | 2008 | Mod2 |
| **Supplement for Muscle Enhancement** | 2016 | 2016 | Mod 1 |
| **Physical Examination** | 2010 | 2011 | Mod 3 |
| **Birth Control** | 2007 | 2013 | Mod 2 |
| **Use of Multivitamins** | 2005 | 2008 | Mod 2 |
| **E-Cigarettes** | 2016 | 2016 | Mod 1 |
| **Tanning Bed Use** | 2010 | 2011 | Mod 1 |
| **Binge Drinking** | 2010 | 2013 | Mod 5 |
| **Marijuana Use** | 2010 | 2013 | Mod 5 |
| **Use of Illegal Drugs** | - | 2010 | Mod 5 |
| **Number of Sex Partners** | - | 2010 | Mod 5 |
| NHS 3 is an open cohort where participants filled out different Mods (order of questionnaire) at different times | | | |

**Supplemental Table 2. Percentages (%) of missing for all variables in Growing Up Today Study (GUTS) and Nurses’ Health Study 3 (NHS3).**

|  | GUTS | NHS3 |
| --- | --- | --- |
| Age | 0 | 0 |
| Sex | 0 | 0 |
| Education | 25 | 12 |
| Race | 0 | 0 |
| Sexual orientation | 1 | 31 |
| Marriage | 1 | 87 |
| Region | 0.1 | 15 |
| Smoking | 31 | 81 |
| BMI | 3 | 0.7 |
| Sleep | 11 | 88 |
| Physical activity | 20 | 4 |
| Diet quality | 11 | 7 |
| Supplements for muscle enhancement | 0 | 0 |
| Physical exam | 0.4 | 0 |
| Birth control | 15 | 46 |
| Multivitamin supplementation | 13 | 0 |
| E-cigarette use | 17 | 97 |
| Tanning bed | 0.6 | 89 |
| Binge drinking | 18 | 38 |
| Marijuana use | 15 | 32 |
| Use of illegal drugs | 0 | 0 |
| Number of persons with sexual contact | 64 | 35 |

**Supplemental Table 3. Odds ratio (OR) of consuming energy drinks characteristics related to intake of energy drinks in Growing Up Today Study (GUTS) and Nurses’ Health Study 3 (NHS3) accounting for missing data.**

|  | GUTS | NHS3 | Pooled |
| --- | --- | --- | --- |
|  | Multivariable-adjusted OR | Multivariable-adjusted OR | Multivariable-adjusted OR |
| **Demographic and Geographical** |  |  |  |
| Age |  |  |  |
| *<25 years* | Ref | Ref | Ref |
| *25-30 years* | 0.95 (0.76, 1.20) | 1.12 (0.85, 1.49) | 0.59 (0.53, 0.67) |
| *30-35 years* | 1.23 (0.37, 4.08) | 0.82 (0.60, 1.13) | 0.55 (0.43, 0.69) |
| *>35 years* | NA | 0.92 (0.69, 1.22) | 0.62 (0.51, 0.75) |
| Sex |  |  |  |
| *Female* | Ref |  | Ref |
| *Male* | 2.86 (2.56, 3.21) | NA | 2.80 (2.51, 3.13) |
| Education |  |  |  |
| *Without a bachelor* | Ref | Ref | Ref |
| *Bachelor* | 1.03 (0.88, 1.21) | 0.63 (0.49, 0.81) | 0.85 (0.75, 0.96) |
| *Master or higher* | 0.72 (0.58, 0.89) | 0.77 (0.52, 1.13) | 0.57 (0.48, 0.68) |
| Race |  |  |  |
| *Non-Hispanic White* | Ref | Ref | Ref |
| *Black* | 3.17 (1.07, 9.35) | 2.34 (1.56, 3.50) | 2.08 (1.44, 3.01) |
| *Hispanic* | 1.40 (0.93, 2.10) | 1.82 (0.95, 3.49) | 1.49 (1.06, 2.11) |
| *Asian* | 0.72 (0.39, 1.35) | 1.05 (0.62, 1.78) | 0.77 (0.52, 1.15) |
| *Other* | 1.08 (0.72, 1.63) | 1.60 (0.83, 3.08) | 1.22 (0.86, 1.72) |
| Sexual orientation |  |  |  |
| *Completely heterosexual* | Ref | Ref | Ref |
| *Bisexual/homosexual* | 0.80 (0.62, 1.05) | 1.94 (1.23, 3.05) | 0.93 (0.74, 1.17) |
| Marriage |  |  |  |
| *Never married* | Ref | Ref | Ref |
| *Married/partner* | 0.92 (0.79, 1.07) | 0.40 (0.30, 0.55) | 0.65 (0.57, 0.75) |
| *Divorced/separated/widowed* | 1.04 (0.54, 2.02) | 0.91 (0.51, 1.64) | 1.44 (0.95, 2.17) |
| Region |  |  |  |
| *Northeast* | Ref | Ref | Ref |
| *Midwest* | 1.06 (0.93, 1.21) | 1.25 (0.91, 1.73) | 1.09 (0.97, 1.23) |
| *South* | 1.14 (0.98, 1.34) | 1.36 (0.99, 1.87) | 1.18 (1.03, 1.35) |
| *West* | 1.11 (0.94, 1.30) | 1.42 (1.01, 1.99) | 1.17 (1.02, 1.35) |
| Lifestyle |  |  |  |
| Smoking |  |  |  |
| *Never smokers* | Ref | Ref | Ref |
| *Current or past smokers* | 2.43 (2.05, 2.88) | 2.71 (2.00, 3.68) | 2.50 (2.17, 2.88) |
| BMI |  |  |  |
| *<25 kg/m^2^* | Ref | Ref | Ref |
| *25-30 kg/m^2^* | 1.33 (1.17, 1.52) | 1.52 (1.23, 1.87) | 1.37 (1.23, 1.53) |
| *≥30 kg/m^2^* | 1.50 (1.25, 1.80) | 2.02 (1.65, 2.48) | 1.74 (1.53, 1.98) |
| Sleep |  |  |  |
| *7-9 hours* | Ref | Ref | Ref |
| *<7 hours* | 1.36 (1.19, 1.55) | 0.92 (0.52, 1.62) | 1.37 (1.21, 1.55) |
| *>9 hours* | 0.83 (0.50, 1.37) | 0.58 (0.42, 0.78) | 0.63 (0.50, 0.81) |
| Physical activity |  |  |  |
| *Low* | Ref | Ref | Ref |
| *Medium* | 1.08 (0.92, 1.25) | 1.01 (0.77, 1.34) | 1.07 (0.94, 1.22) |
| *High* | 1.13 (0.96, 1.32) | 1.65 (1.28, 2.12) | 1.28 (1.12, 1.46) |
| Diet quality |  |  |  |
| *Low* | Ref | Ref | Ref |
| *Medium* | 0.77 (0.67, 0.89) | 0.78 (0.54, 1.12) | 0.77 (0.68, 0.88) |
| *High* | 0.73 (0.63, 0.84) | 0.82 (0.57, 1.17) | 0.71 (0.62, 0.81) |
| Supplements for muscle enhancement |  |  |  |
| *No* | Ref | Ref | Ref |
| *Yes* | 1.13 (0.93, 1.36) | 1.66 (1.04, 2.64) | 1.27 (1.07, 1.51) |
| Physical exam in the past 1-2 years |  |  |  |
| *Yes* | Ref | Ref | Ref |
| *No* | 1.11 (0.96, 1.27) | 1.53 (1.29, 1.82) | 1.40 (1.26, 1.56) |
| Birth control, female participants only |  |  |  |
| *No* | Ref | Ref | Ref |
| *Yes* | 1.25 (1.04, 1.51) | 1.35 (1.06, 1.73) | 1.28 (1.11, 1.49) |
| Multi-vitamin supplementation |  |  |  |
| *No* | Ref | Ref | Ref |
| *Yes* | 0.93 (0.83, 1.04) | 8.13 (6.18, 10.71) | 1.22 (1.10, 1.35) |
| **Risky behaviours** |  |  |  |
| E-cigarette use |  |  |  |
| *No* | Ref | Ref | Ref |
| *Yes* | 2.12 (1.64, 2.75) | 9.87 (6.68, 14.59) | 4.56 (3.63, 5.74) |
| Tanning bed |  |  |  |
| *No* | Ref | Ref | Ref |
| *Yes* | 2.50 (2.18, 2.85) | 0.73 (0.52, 1.02) | 2.37 (2.11, 2.67) |
| Binge drinking |  |  |  |
| *No* | Ref | Ref | Ref |
| *Yes* | 1.43 (1.26, 1.62) | 1.44 (1.17, 1.78) | 1.23 (1.11, 1.36) |
| Marijuana use |  |  |  |
| *No* | Ref | Ref | Ref |
| *Yes* | 1.51 (1.32, 1.72) | 0.54 (0.43, 0.68) | 1.04 (0.93, 1.16) |
| Use of illegal drugs |  |  |  |
| *No* | Ref | Ref | Ref |
| *Yes* | 1.37 (1.10, 1.71) | 1.82 (1.32, 2.51) | 1.49 (1.25, 1.77) |
| Number of persons with sexual contact |  |  |  |
| 1 person | Ref | Ref | Ref |
| 2 persons | 1.04 (0.67, 1.62) | 1.67 (0.79, 3.53) | 1.18 (0.81, 1.72) |
| 3-5 persons | 1.32 (0.89, 1.94) | 1.60 (0.86, 2.98) | 1.35 (0.98, 1.86) |
| >5 persons | 1.77 (1.19, 2.64) | 4.24 (2.41, 7.45) | 2.50 (1.83, 3.41) |

We imputed missing values as the value that has the highest percentage for variables that has skewed distributions, i.e., ‘no’ for smoking tatus, use of tanning bed, binge drinking, marijuana use, and multivitamin use, married/partner for marriage status, and completely heterosexual for sexual orientation. We fitted multivariable logistic regression including those imputed variables to obtain OR of these variables with energy drink intake, and then added the rest of the variables into the multivariable model one by one to obtain OR of that variable.

In pooled analysis, we combined individual data in NHS3 and GUTS into one dataset and conducted regression analyses in the pooled dataset.
